# Supplementary material for: Effectiveness of Antenatal Corticosteroids in reducing morbidities and mortality in Preterm neonates: Evidence from a Tertiary Level Hospital in Nepal
Source: PLoS One. 2025 Oct 24;20(10):e0334318. doi: 10.1371/journal.pone.0334318 (PMC12551915; doi:10.1371/journal.pone.0334318)
Supplement: Attachment — Submitted filename: Response to Reviewers.docx [file pone.0334318.s003.docx]

**Response to Reviewers and Editor**

Dear Editor,

I am thankful to you for your thoughtful review of my article entitled ‘Effectiveness of Antenatal Corticosteroids in reducing morbidities and mortality in Preterm neonates: Evidence from a Tertiary Level Hospital in Nepal’’ and providing an opportunity to revise and resubmit it. I am grateful to you and for the time and effort you dedicated for this evaluation.

Based on the feedback you have provided; I have made the necessary revision as required. I have attached herewith the key changes which I made as per the review along with revised version of the manuscript.

**Editor comments:** The manuscript titled “Effectiveness of Antenatal Corticosteroids in Reducing Morbidities and Mortality in Preterm Neonates: Evidence from a Tertiary Level Hospital in Nepal” presents important findings on the use of antenatal corticosteroids (ACS) in a resource-limited setting. Reviewer 1 raised key concerns regarding clarity on the study design (randomization and ethical considerations), missing information on timing of steroid administration, and minor typographical errors in the results section. Reviewer 2 acknowledged the significance of the study and its potential impact but recommended improving the presentation of statistical data (e.g., consistent reporting of 95% CIs) and addressing possible confounders such as maternal subclinical infections. Addressing these methodological and editorial points will enhance the scientific quality and clarity of the manuscript.

Response: Thank you for the comments. We have now addressed all of the points raised in the manuscript.

**Reviewer #1 comments**

Comment 1-It is not clear how the two groups were chosen. Is this a randomised study? And if so, can this procedure be considered ethically correct?

Response: This is not a randomized study. We have specifically mentioned this in line 95 and 96 “This was an observational study and no random assignment of ACS was done; all decisions were made by treating physicians”.

Comment 2- The time between the course of corticosteroids and delivery was not given.

Response: We acknowledge this as a limitation of the study as we could not collect the data on the time interval.

Comment 3- the 3rd paragraph of the results table 2 instead of table 1

Response: We have now corrected this in the result section with table 2.

Comment 4- the 3rd line of the results lacks a space between POG. and 206

Response: We have now corrected this typographical error.

**Reviewer #2**:

Comment 1-The evidence of the effectiveness of antenatal steroids in high resource setting is undebatable, yet the data for their use in limited resource settings is limited. Here the authors have presented a compelling and needed study supporting the use of antenatal steroid use in a limited resource setting.

Response: Thank you for this thoughtful comment. We appreciate your acknowledgement of the importance and relevance of our study.

Comment 2-

Introduction:

SDG target - please write out any acronyms in full on first use

POG - please write out any acronyms in full on first use

Response: We have revised this as suggested

Comment 3-

Results:

The authors state in the results sections that "distribution of outcomes for both groups, p-values indicating the statistical significance, and adjusted odds ratios (aOR) with confidence intervals (95% CI) for each outcome". When reporting the results in text, there is a lot of repetition to the tables. The authors also do not report 95% CIs in text, but just state 95% CI which makes the results very cluttered and difficult to read, e.g. "administration of ACS is significantly associated with the development of RDS 38.2% (58) vs 21.8% (45), (p-value=<0.0001) and (aOR= 4.181, 95% CI). I would suggest simply reporting percentages and p-values, or just p-values and reference tables reduce confusion and repitition. Otherwise please include the actual 95% CI values in text and not simply state "aOR= ..., 95% CI".

Response: We have revised this clearly referring to the tables reducing confusion and repetition.

Comment 4-

There was a greater number of preterm infants who did not receive ACS that had neonatal sepsis compared to preterm infants that received ACS. Is this due to a greater number developing infection after birth in non-ACS infants compared to preterm infants whose received ACS? Or were there potentially a greater number of non-ACS mothers having sub-clinical bacterial infections that they passed on to their infants, and this lead to sepsis postnatally? Similarly were there a higher number of mothers that had overt infection and received prenatal antibiotics prior to preterm birth that could have influenced this outcome? Subclinical infection in mothers can be a risk factor for preterm birth as well as RSD, could this be a confounder in the outcomes of the study?

Response: We acknowledge this could be a potential confounder, which could be assessed as this data were not collected
